# Oral delivery of the intracellular domain of the insulinoma-associated protein 2 (IA-2ic) by bacterium-like particles (BLPs) prevents type 1 diabetes mellitus in NOD mice

**DOI:** 10.1080/10717544.2022.2053760

**Published:** 2022-03-21

**Authors:** Ruifeng Mao, Menglan Yang, Rui Yang, Yingying Chen, Enjie Diao, Tong Zhang, Dengchao Li, Xin Chang, Zhenjing Chi, Yefu Wang

**Affiliations:** a School of Life Sciences, Jiangsu Collaborative Innovation Center of Regional Modern Agriculture & Environmental Protection, Huaiyin Normal University, Huai’an, China; b Nanjing Lishui People’s Hospital, Zhongda Hospital Lishui Branch, Southeast University, Nanjing, China; c Huai’an First People’s Hospital, Nanjing Medical University, Huai’an, China; d State Key Laboratory of Virology, College of Life Sciences, Wuhan University, Wuhan, China

**Keywords:** Type 1 diabetes mellitus, insulinoma-associated protein 2, bacterium-like particles, antigen delivery, oral tolerance

## Abstract

Antigen-specific immune tolerance, which possesses great potential in preventing or curing type 1 diabetes mellitus (T1DM), can be induced by oral vaccination with T1DM-related autoantigens. However, direct administration of autoantigens via oral route exhibits a low tolerance-inducing effect as a result of the digestion of protein antigens in the gastrointestinal tract (GIT) and therefore, a large dosage of autoantigens may be needed. In this study, bacterium-like particles (BLPs) made from food-grade lactic acid bacteria were used to deliver the intracellular domain of the insulinoma-associated protein 2 (IA-2ic). For this purpose, BLPs-IA-2ic vaccine in which IA-2ic bound to the surface of BLPs was constructed. BLPs enhanced the stability of the delivered IA-2ic based on the stability analysis *in vitro*. Oral administration of BLPs-IA-2ic significantly reduced T1DM incidence in NOD mice. The mice fed BLPs-IA-2ic exhibited a significant reduction in insulitis and preserved the ability to secrete insulin. Immunologic analysis showed that oral vaccination with BLPs-IA-2ic induced antigen-specific T cell tolerance. The results revealed that the successful induction of immune tolerance was dependent on the immune deviation (in favor of T helper 2 responses) and CD4^+^CD25^+^FoxP3^+^ regulatory T cells. Hence, oral vaccination with BLPs-IA-2ic shows potential for application in preventing T1DM.

## 1. Introduction

Apart from preventing invasion of pathogens, the immune response plays an important role in preventing reactivity to self-antigens, thereby preventing the development of autoimmunity by complicated suppression tolerance mechanisms. Immune tolerance, including central and peripheral tolerance, relies on complicated clonal deletion/anergy of T and B cells as well as suppression by regulatory immune cells (Issa & Wood, 2012; Wambre & Jeong, 2018). Tolerance induction can be achieved by introducing proteins or peptides through various methods, such as intravenous injection, intranasal administration, skin administration as well as oral administration (Wang & Tisch, 2008; Xu et al., 2013; Mao et al., 2020a, 2020b). As a result of its ability to induce systemic unresponsiveness to orally administered antigens and its non-invasiveness, oral tolerance has been intensively investigated (Sricharunrat et al., 2018). Most of these oral tolerance studies performed in animals and human clinical trials aim to prevent and treat allergies, transplantation rejection as well as autoimmune disorders, including type 1 diabetes mellitus (T1DM).

T1DM is characterized by hyperglycemia resulting from insulin deficiency, which is caused by the autoimmune attack of pancreatic β-cell mediated by T cell. Once diagnosed, patients with T1DM require lifelong insulin therapy. It has been suggested that a complex interplay between genetic factors and environmental factors as well as microbiome, metabolism is necessary; however, the detailed mechanism that triggers this autoimmune process remains to be elucidated (DiMeglio et al., 2018; Chwalba et al., 2021). Until now, the classically studied autoantigens associated with T1DM include (pro)insulin, glutamate decarboxylase 65 (GAD65), insulinoma-associated protein 2 (islet antigen 2, IA-2), and zinc transporter 8 (ZNT8). In recent years, several additional candidates such as tetraspanin-7 (McLaughlin et al., 2016) and chromogranin A (Li et al., 2015) have been identified and added to this list. The appearance of antibodies against specific autoantigens seems to be markers of pancreatic β-cell destruction, rather than be its cause. According to the related symptoms and the number of islet-directed autoantibodies, three stages have been suggested during the natural progression of T1DM in individuals at high genetic risk (DiMeglio et al., 2018; Powers, 2021). In the first stage, two or more T1DM-related autoantibodies can be detected. However, the blood sugar concentration is normal. In the second stage, the autoantibodies are accompanied by dysglycemia without symptoms. In the third stage, T1DM is clinical diagnosed and insulin therapy should be initiated. It is noted that autoantibodies occur before the clinical onset of T1DM and this progression reflected by β-cell loss could take months or years. Thus, as a predictable disease (Simmons & Michels, 2015), the T1DM vaccine may be designed and applied to prevent and/or interrupt the autoimmune response against the above T1DM-related autoantigens by reinstating immunological tolerance to them, and finally, preventing and/or reversing T1DM (Desai et al., 2021). For this purpose, the above-mentioned oral tolerance therapy may represent a promising strategy.

In order to enhance the oral bioavailability of peptide/protein drugs, delivery vehicles that can protect them from digestion in the gastrointestinal tract (GIT) are needed. As a result of their immunostimulating properties, inanimate bacterium-like particles (BLPs), which can be obtained from natural lactic acid bacteria (LAB), have been applied as immunostimulants to enhance the protective efficiency of existing vaccine. Furthermore, they can also be used as microparticulate systems to deliver peptide/protein antigens (Mao et al., 2016; Wong et al., 2018). BLPs-SCI-59 vaccine, which contains a single-chain insulin (SCI-59) analog, could successfully prevent autoimmune diabetes in NOD mice by inducing antigen-specific immune tolerance (Mao et al., 2017, 2019). It has been shown that combinatorial therapeutic strategies, including oral administration of different autoantigens, exhibit promising effectiveness in inducing oral tolerance in T1DM (Mao et al., 2020a, 2020b).

Unlike the major autoantigen-(pro)insulin, the role of GAD65 and IA-2 in initiating T1DM in NOD mice remains controversial as a result of the knockout studies and the corresponding autoantibodies analysis (Bonifacio et al., 2001; Lieberman & DiLorenzo, 2003; Kubosaki et al., 2004; DiLorenzo, 2011). However, T-cell reactivity to GAD65 and IA-2 plays a key role in early activating β-cell specific T cells that are specific for other antigens and this mediates β-cell damage (DiLorenzo, 2011). The ability of GAD65 to induce oral immune tolerance has been widely tested and its positive effect in preventing or reversing T1DM by inducing antigen-specific oral tolerance in NOD mice has been detected (Mao et al., 2020a, 2020b). In addition, DNA vaccination encoding IA-2 can prevent the onset and development of T1DM in NOD mice and an immune tolerance which may be caused by IA-2 overexpression was detected (Guan et al., 2012). Therefore, as a preliminary study for further evaluation of the bivalent BLPs vaccine containing SCI-59 and IA-2 in preventing T1DM, our present study constructed LAB BLPs-IA-2ic vaccine containing the intracellular domain of IA-2 (residues 604-979, IA-2ic) and assessed whether this monovalent vaccine possesses the ability to induce oral immune tolerance to suppress T1DM in animals.

## 2. Materials and methods

### 2.1. Plasmid, strains, and mice

In order to obtain the IA-2ic-3LysM fusion protein in which the anchor domain LysM repeats contained in the major autolysin AcmA (GenBank accession number: U17696.1) is fused to the C-terminus of IA-2ic, the codon-optimized DNA sequence (GenBank accession number: OL961563) encoding this fusion protein was synthesized and ligated into the pET20b(+) vector, yielding the recombinant expression plasmid pET20b-IA-2ic-3L (Sangon Biotech, Shanghai, China). *Escherichia coli* BL21 (DE3) and *Lactococcus lactis* MG1363 were stored in our lab. *E. coli* BL21 (DE3) was routinely cultured aerobically at 37 °C in LB medium. *L. lactis* MG1363 was cultured statically in GM17 medium containing 0.5% glucose. Female NOD/LtJ mice (4 weeks old), which were purchased from Slaccas (Slaccas Laboratory Animal, Shanghai, China), were housed in pathogen-free conditions with standard chow as well as sterilized water. The study protocol was approved by the Animal Experimental Ethics Committee of Huaiyin Normal University.

### 2.2. Expression of the IA-2ic-3LysM fusion protein

The recombinant expression plasmid pET20b-IA-2ic-3L was transformed in BL21 (DE3) competent cells using the standard CaCl_2_-heat shock method. Successfully transformed BL21 (DE3) cells were then cultured overnight in LB medium with 100 μg/mL ampicillin. Cultures were diluted 1:20 into 100 mL fresh LB medium and cultured until their OD_600_ reach 0.6. In order to induce the expression of IA-2ic-3LysM fusion protein, 0.5 mM isopropyl-β-d-thiogalactopyranoside (IPTG, Sangon Biotech, Shanghai, China) was added, and then cultures were further grown for another 12 h at 20 °C with shaking at 300 rpm.

### 2.3. Identification and purification of recombinant protein

After expression, *E. coli* cells were harvested by centrifugation. Lysis buffer (50 nM Tris–HCl, 0.05 mg/mL lysozyme, 1 mM PMSF, pH 7.4) was added to resuspend the cell pellet, yielding a 10% (w/v) suspension. After overnight incubation at −20 °C, sonication was applied, and the supernatant was collected. Based on the confirmation of successful expression of the IA-2ic-3LysM fusion protein by 10% SDS-PAGE and Western blot using the monoclonal anti His-tag antibody (Sangon Biotech, Shanghai, China) as the primary antibody (1:2000 dilution), purification of the IA-2ic-3LysM fusion protein from the harvested sample was performed using a Ni-NTA column (Thermo Fisher, Waltham, MA) following the manufacturer's instructions. Eluted IA-2ic-3LysM fusion protein was pooled and dialyzed against phosphate buffer saline (PBS, pH 7.0), and then concentrated using a 30-kDa cutoff filter (Millipore, Billerica, MA). Protein concentration was analyzed via Bradford Protein Assay (Bio-Rad, Hercules, CA).

### 2.4. Production of BLPs-IA-2ic vaccine

0.3 volume PBS was used to wash the harvested stationary-phase *L. lactis* MG1363 cells. After washing two times, cell pellets were boiled for 30 min in 0.2 volume 10% trichloroacetic acid (TCA). After vigorous washing, the obtained MG1363 BLPs were resuspended in PBS (2.5 × 10^10^ BLPs/mL). Different amounts of purified IA-2ic-3LysM fusion protein (from 60 to 150 μg at 10 μg intervals) were added to 2.5 × 10^9^ BLPs in 1 mL PBS, and samples were then incubated for 60 min on an end-over-end rotator at room temperature. After binding, MG1363 BLPs-IA-2ic were harvested by centrifugation and the supernatant was collected and saved for Western blot to analyze whether there was some IA-2ic-3LysM residue in the supernatant after binding. The harvested pellets were washed twice with PBS and finally resuspended in 100 μL PBS. Using the monoclonal anti His-tag antibody and FITC-conjugated IgG secondary antibody (Sangon Biotech, Shanghai, China), immunofluorescence microscopy was applied as previously described (Mao et al., 2017) to confirm the successful binding of IA-2ic-3LysM on the surface of BLPs.

### 2.5. Stability of bound protein

As described previously, simulated gastric juice (pH 2.0 or 4.0), which contains 10 U/mL pepsin (Sangon Biotech, Shanghai, China), was prepared (Charteris et al., 1998). 2.5 × 10^9^ BLPs (100 μL), 100 μg free IA-2ic-3LysM fusion protein (100 μL), and 100 μL BLPs-IA-2ic suspension containing 100 μg IA-2ic-3LysM fusion protein were mixed with 100 μL simulated gastric juice (pH 2.0 or 4.0), respectively. Then, the mixture was incubated at 37 °C for 15 min. At 0, 3, 6, 9, 12, and 15 min, samples (10 μL) were taken from each mixture and subjected to detection of IA-2ic-3LysM fusion protein by ELISA. Briefly, samples were diluted 10-fold in PBS and added into ELISA plates, which were coated overnight at 4 °C. After washing with PBS containing 0.05% Tween-20 (PBST), 3% bovine serum albumin (BSA) in PBS was added to the plates and then incubated for 3 h at 37 °C. After washing, 100 μL anti-His monoclonal antibody (1:1000 dilution) was added and incubated for 2 h at 37 °C. After washing, 100 μL horseradish peroxidase (HRP)-conjugated secondary antibody was added and incubated for 1 h at 37 °C. After washing, 100 μL TMB substrate (Sangon Biotech, Shanghai, China) was added and incubated for 10 min at 37 °C before absorbance at 450 nm (OD_450_) was measured.

At different temperatures (–20 °C, 4 °C and room temperature), tubes containing 2.5 × 10^9^ BLPs (100 μL), 100 μg free IA-2ic-3LysM fusion protein (100 μL) and 100 μL BLPs-IA-2ic suspension containing 100 μg IA-2ic-3LysM fusion protein were stored for 60 days. At 1, 3, 5, 10, 20, 40, and 60 days, tubes containing different samples at different temperatures were taken and subjected to ELISA analysis as described above.

### 2.6. Vaccination and estimate of autoimmune diabetes

Forty-five mice (4-week-old) were randomly sorted into three groups consisting of 15 mice each. Beginning at 5 weeks of age, the three groups of mice were gavaged with 2.5 × 10^9^ BLPs (100 μL), 100 μg free IA-2ic-3LysM fusion protein (100 μL), and 100 μL BLPs-IA-2ic suspension containing 100 μg IA-2ic-3LysM fusion protein, respectively, yielding the corresponding BLPs group, IA-2ic-3LysM group and BLPs-IA-2ic group. The gavage was performed once a day during the first week and subsequently three times per week in the following 15 weeks. Mouse serum was harvested from blood samples collected from the orbit every four weeks and stored at −80 °C. Mouse body weight as well as blood glucose was assessed weekly. Diabetes could be diagnosed when mouse blood glucose levels exceed 16 mmol/L for two consecutive weeks as well as it presented with the related symptoms, such as polyuria and weight loss.

### 2.7. Serum antibody analysis

At the end of dosing cycle (20-week-old), analysis of anti-IA-2 antibodies in mouse serum was performed by ELISA. After blocking with IA-2 protein (Sangon Biotech, Shanghai, China), serum samples were added to the plate and incubated. HRP-tagged secondary antibodies (Sangon Biotech, Shanghai, China) including anti-mouse IgG, IgG1, and IgG2a antibody were used. The titer was defined as previously (Liu et al., 2016) and individually determined for each sample based on the measurement of OD_450_.

### 2.8. Serum C-peptide analysis and pancreas histopathology

During the whole observation period, serum C-peptide levels at different time points were measured with an ELISA kit (Sigma, St. Louis, MO) following the manufacturer’s recommended protocol. Performed as described previously (Lang et al., 2017), surviving mice in all three groups were euthanized when the observation period ended (40-week-old), and pancreas were collected for hematoxylin–eosin (HE) staining to evaluate insulitis. Based on the level of immune infiltration, islets were divided into four categories, including no infiltration (1), peri-insulitis (2), mild insulitis (3), and severe insulitis (4). For each mouse in each group, at least 20 islets were applied for insulitis analysis.

### 2.9. Splenocytes proliferation assay and cytokine-specific ELISA

Splenocytes of the above euthanized mice in each group were isolated, diluted and incubated in 96-well plates at 1 × 10^6^ cells/well. Analyzed in quadruplicate, medium, 10 μg/mL BSA, 2.5 μg/mL concanavalin A (Con A, Invitrogen, Carlsbad, CA), or 10 μg/mL IA-2 protein (Sangon Biotech, Shanghai, China) was added to stimulate splenocytes. After 48 h of incubation, proliferation was determined based on WST-8 test (Beyotime, Nantong, China) at 570 nm (reference 630 nm) with Microplate reader (Bio-Rad, Hercules, CA). The proliferative response level was presented with a stimulation index (SI), which was calculated as the ratio of the average absorption values of samples stimulated with different antigens to those stimulated with medium alone. Splenocytes were isolated, cultured (1 × 10^6^ cells/well) and stimulated with IA-2 as described above. After 48 h of incubation, the harvested supernatants were used for cytokine analysis by ELISA (Beyotime, Nantong, China). Performed following the product manual, the levels of five different cytokines, including interleukin (IL)-2, IL-4, IL-10, and interferon (IFN)-γ as well as transforming growth factor (TGF)-β, were simultaneously measured. Each sample was analyzed in triplicate.

### 2.10. Analysis of regulatory T cells (Tregs)

Pancreatic lymph nodes (PLNs), which were collected from the above euthanized mice, were subjected to lymphocytes isolation with the mouse lymphocyte separation kit (Solarbio, Beijing, China). According to the manufacturer’s recommended protocol, the harvested lymphocytes were stained for Tregs (CD4^+^CD25^+^FoxP3^+^) using the FoxP3 staining kit from Invitrogen (Carlsbad, CA), and then subjected to analysis on Accuri C6 plus flow cytometer equipped with Accuri C6 Plus software (BD Biosciences, Franklin Lakes, NJ).

### 2.11. Statistical analysis

Mantel-Cox log-rank test was applied to analyze significant difference of T1DM incidence among different groups. Data are presented as means ± SD. Comparisons between experimental groups were analyzed with the help of ANOVA followed by Student’s *t*-test. Statistical significance is indicated by *p < .05; **p < .01.

## 3. Results

### 3.1. Production of IA-2ic-3LysM in *E. coli*

**Figure 1.**
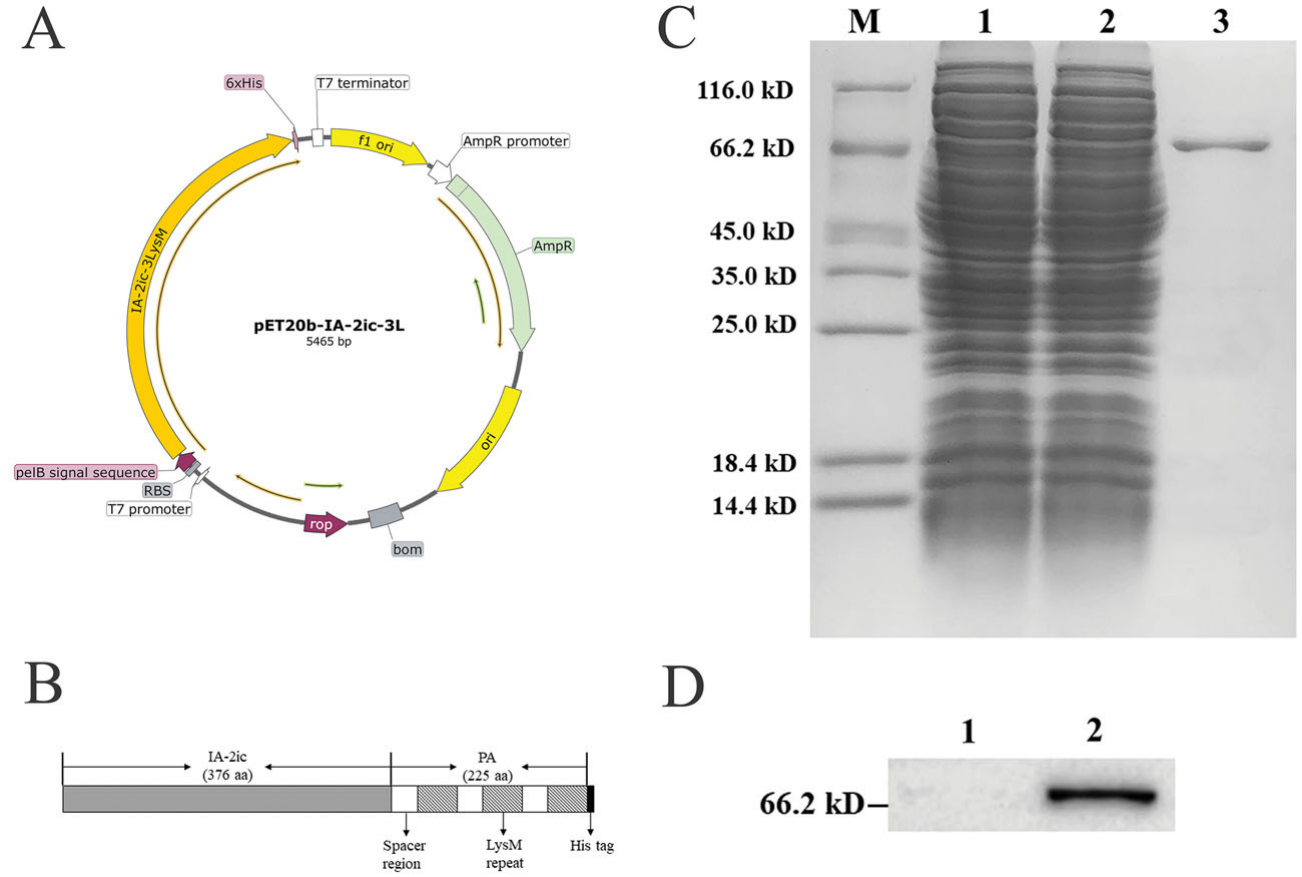
Characteristic and identification of recombinant IA-2ic-3LysM fusion protein. (A) Schematic representation of recombinant plasmid pET20b-IA-2ic-3L. (B) Schematic representation of IA-2ic-3LysM fusion protein. Protein anchor (PA) containing 3LysM repeats was fused to the C-terminus of IA-2ic. (C) SDS-PAGE analysis: lane M, protein standard; lane 1, supernatant of IPTG-induced lysate (*E. coli* BL21 pET20b-IA-2ic-3L) by sonication; lane 2, flow-through of Ni-NTA column; lane 3, elute of Ni-NTA column containing IA-2ic-3LysM. (D) Western blot analysis: lane 1, blank control; lane 2, purified IA-2ic-3LysM fusion protein was detected by Western blotting using anti-His monoclonal antibody.

In order to express IA-2ic-3LysM fusion protein in *E. coli*, recombinant pET20b-IA-2ic-3L plasmid (Figure 1(A)) was constructed. As shown in Figure 1(B), protein anchor (PA, 225 amino acids) containing three LysM motifs and spacer regions was fused to the C-terminus of mouse IA-2ic (376 amino acids). Based on induction by IPTG at 20 °C, IA-2ic-3LysM fusion protein was successfully detected in soluble cell lysates prepared by sonication (data not shown). After purification, a 66 kDa band, which is close to the predicted molecular weight of IA-2ic-3LysM, was detected by SDS-PAGE (Figure 1(C)) and Western blot (Figure 1(D)). In addition, IA-2ic-3LysM fusion protein was obtained with a yield of 46 mg/L fermentation media under the induction condition used in this study (under 0.5 mM IPTG induction at 20 °C for 12 h with shaking at 300 rpm).

### 3.2. Binding of IA-2ic-3LysM to BLPs

**Figure 2.**
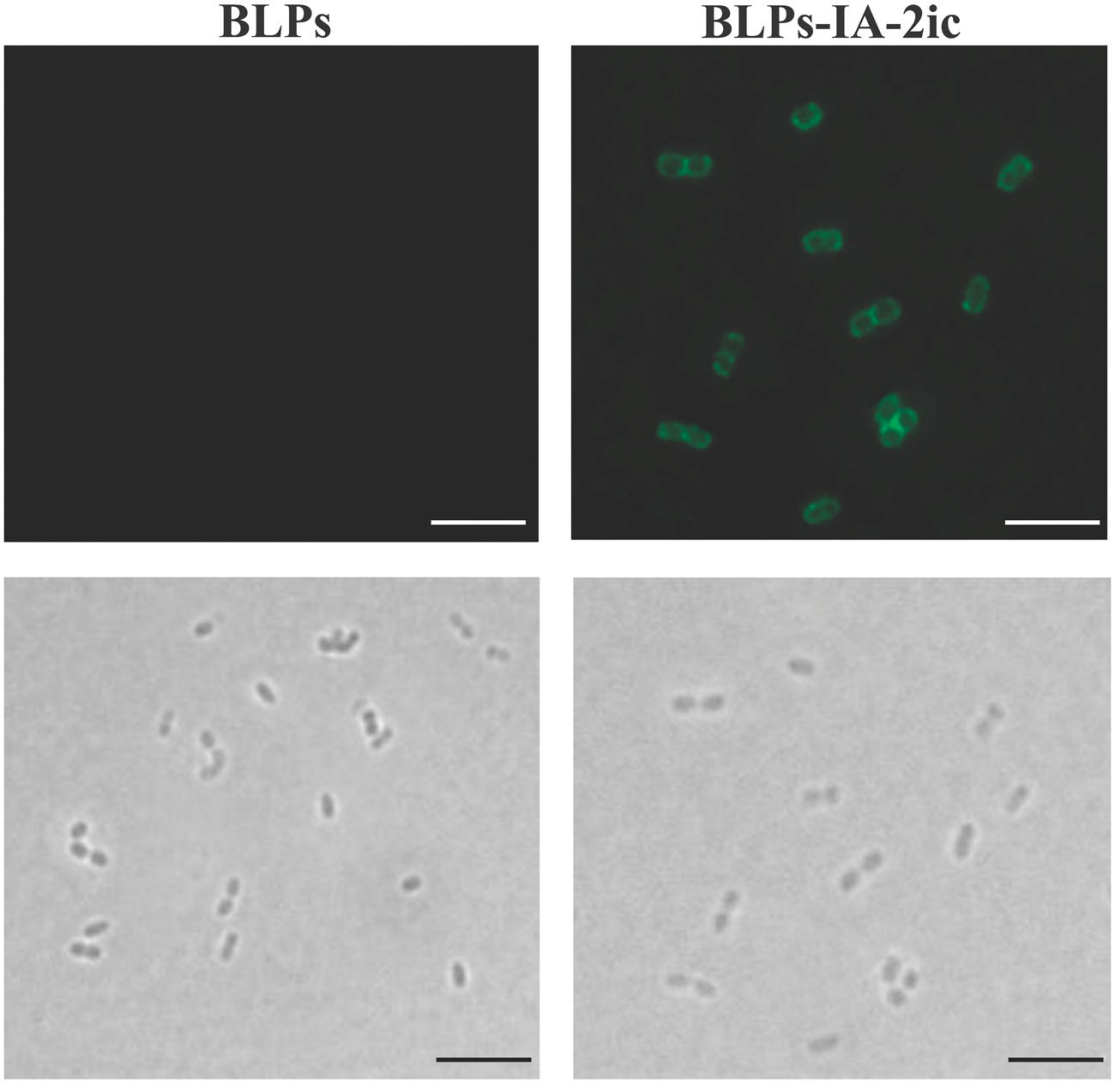
Analysis of BLPs-IA-2ic by immunofluorescence microscopy. Immunofluorescence (top) and bright-field microscopy images (bottom) of BLPs alone and BLPs-IA-2ic. Images were acquired at ×100 magnification on an Olympus Fluoview IX70 confocal laser scanning microscope. Scale bar: 5 μm.

Obtained from the living *L. lactis* MG1363 cells, 2.5 × 10^9^ BLPs were incubated with different amounts of IA-2ic-3LysM fusion protein (60–150 μg). After incubation, Western blot was applied to assess the binding efficiency and it was observed that all these fusion proteins in groups containing 60–100 μg IA-2ic-3LysM fusion protein successfully bound to the surface of MG1363 BLPs, as no IA-2ic-3LysM fusion protein was detected in the supernatant after binding. However, some IA-2ic-3LysM remained in the supernatant fraction after binding in groups containing 110–150 μg IA-2ic-3LysM fusion protein (data not shown). Therefore, these results suggested that at most 100 μg IA-2ic-3LysM fusion protein can bind to 2.5 × 10^9^ MG 1363 BLPs under the conditions used in this study. As shown in Figure 2, this successful binding was further verified by immunofluorescence microscopy. Therefore, BLPs-IA-2ic vaccine can be obtained successfully.

### 3.3. Blps-IA-2ic vaccine stability *in vitro*

**Figure 3.**
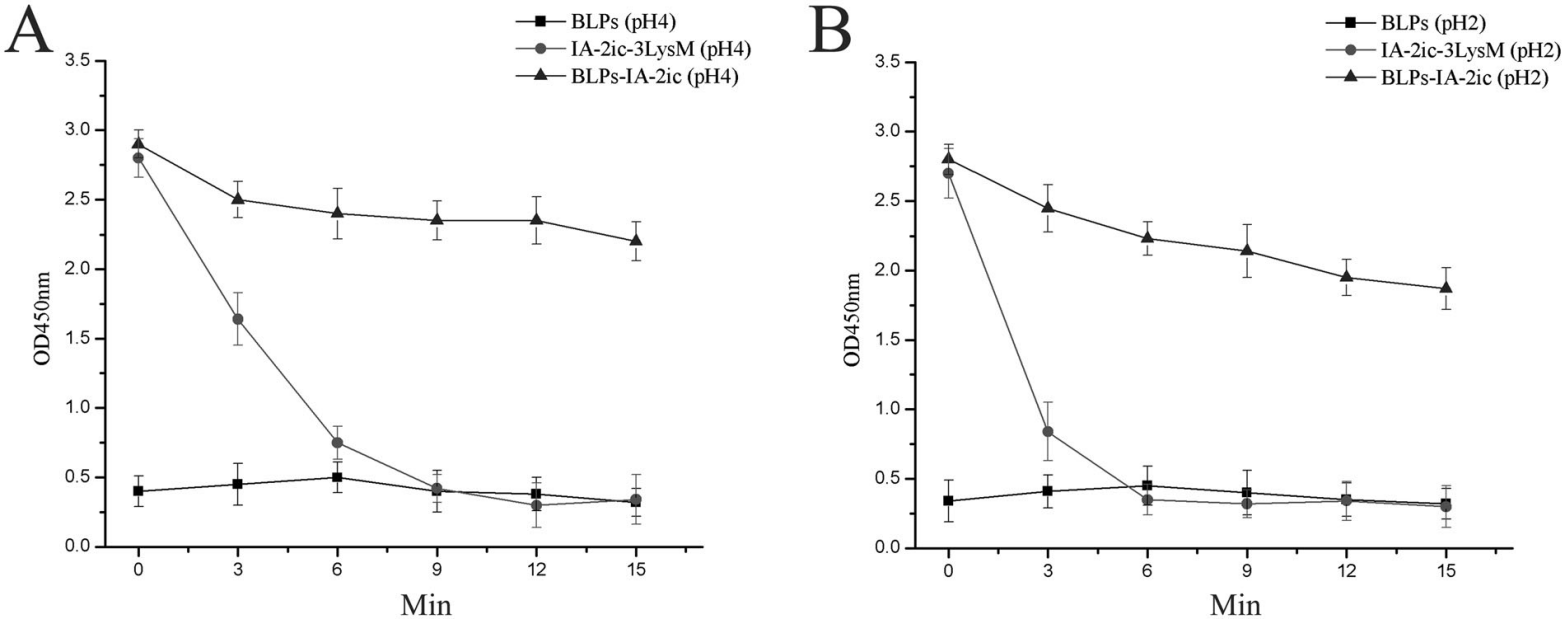
Stability analysis of BLPs-IA-2ic in the stimulated gastric juice. BLPs alone, free IA-2ic-LysM, and BLPs-IA-2ic were incubated with stimulated gastric juice with pH 4.0 (A) and pH 2.0 (B), respectively, and at indicated time, samples were subjected to analysis using ELISA. Data are shown as means ± SD.

To evaluate the stability of the constructed BLPs-IA-2ic vaccine, simulated gastric juice with two different pH values (pH 2.0 or 4.0) was first applied. As shown in Figure 3, free IA-2ic-3LysM fusion protein disappeared rapidly as there was almost no detectable protein at 9 min (pH 4.0) or at 6 min (pH 2.0). Oppositely, IA-2ic-3LysM bound to BLPs (BLPs-IA-2ic group) exhibited a better stability in the same simulated juice.

**Figure 4.**
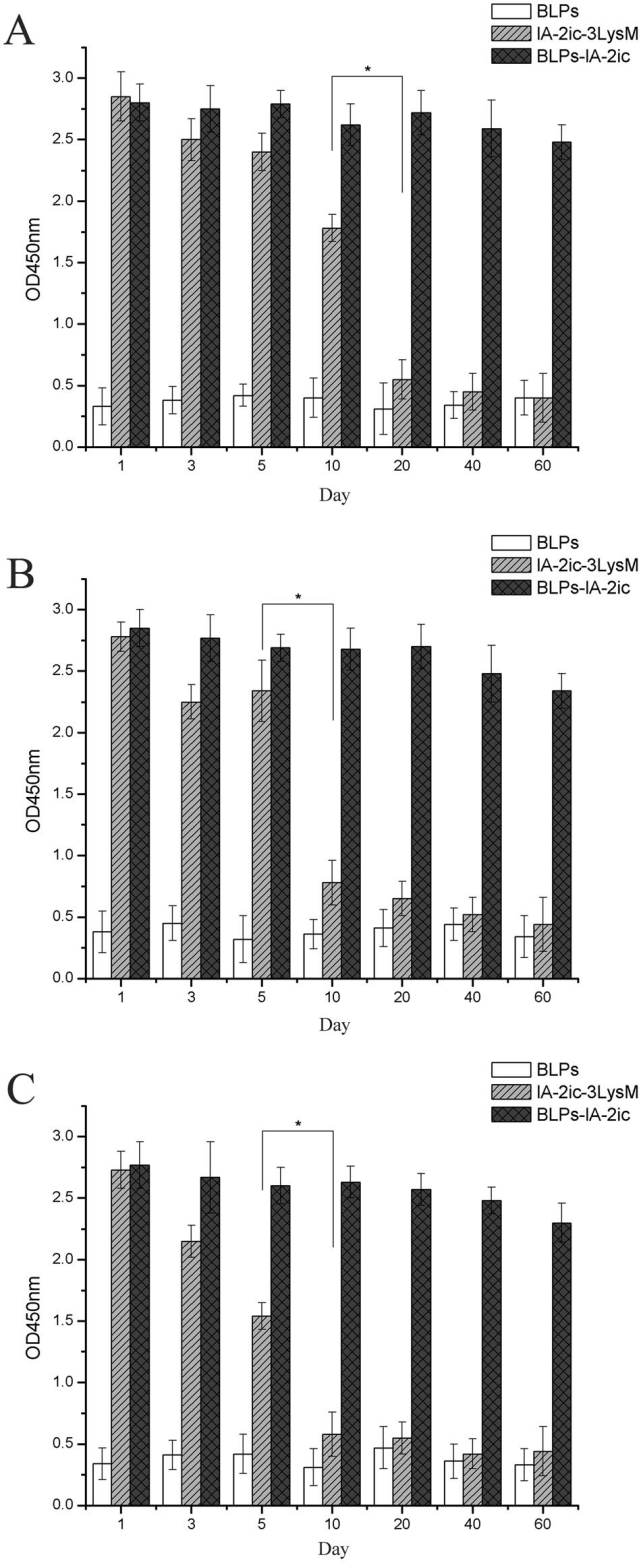
Stability analysis of BLPs-IA-2ic at different temperatures. At –20 °C (A), 4 °C (B) and room temperature (C), BLPs, free IA-2ic-3LysM, and BLPs-IA-2ic were stored for 60 days, and at indicated time, samples were subjected to analysis using ELISA. Data are shown as means ± SD. *p < .05.

Second, the stability of bound IA-2ic-3LysM over time was evaluated at different temperatures (–20 °C, 4 °C and room temperature). Significant degradation of free IA-2ic-3LysM fusion protein was observed on day 20, day 10, and day 10 under −20 °C, 4 °C and room temperature, respectively (Figure 4). However, no significant degradation of IA-2ic-3LysM bound to BLPs (BLPs-IA-2ic group) was observed in a 60 days period regardless of the temperature (Figure 4).

### 3.4. Prevention of autoimmune diabetes in NOD mice by oral vaccination with BLPs-IA-2ic

**Figure 5.**
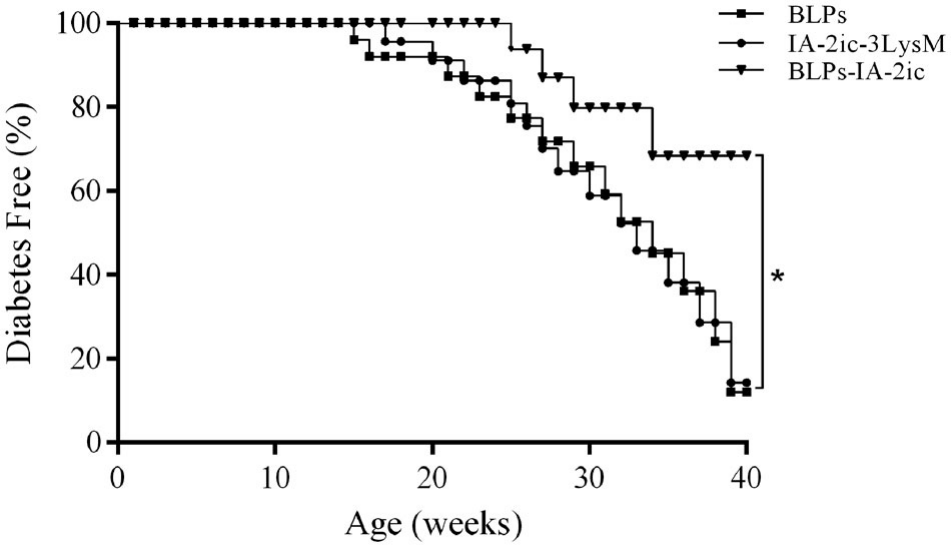
Suppression of diabetes by oral vaccination with BLPs-IA-2ic. Mice were fed BLPs (*n* = 15), IA-2ic-3LysM (*n* = 15), or BLPs-IA-2ic (*n* = 15) once daily during the first week and then three times per week in the following 15 weeks. Diabetes was diagnosed when mouse blood glucose levels were higher than 16 mmol/L for two consecutive weeks as well as it presented with the related symptoms, such as polyuria and weight loss. *p < .05.

Based on the detection of hyperglycemia and related symptoms, incidence of autoimmune diabetes in all groups was recorded and analyzed (Figure 5). T1DM onset was delayed in the BLPs-IA-2ic group (25-week-old), as compared to the BLPs group (15-week-old) and IA-2ic-3LysM group (17-week-old). When the observation period ended (40-week-old), the BLPs group and IA-2ic-3LysM group exhibited a similar diabetes incidence (13 out of 15 mice, 87%). However, a significant reduction in diabetes incidence was observed in BLPs-IA-2ic group (five out of 15 mice, 33%, *p* < .05). In addition, at the end of the observation period (40-week-old), the survival rate in BLPs group, IA-2ic-3LysM group and BLPs-IA-2ic group was 27% (4/15), 40% (6/15), and 87% (13/15), respectively. These results indicate that T1DM was successfully prevented by oral vaccination with BLPs-IA-2ic.

### 3.5. Reduction of insulitis and preservation of C-peptide secretion by oral vaccination with BLPs-IA-2ic

**Figure 6.**
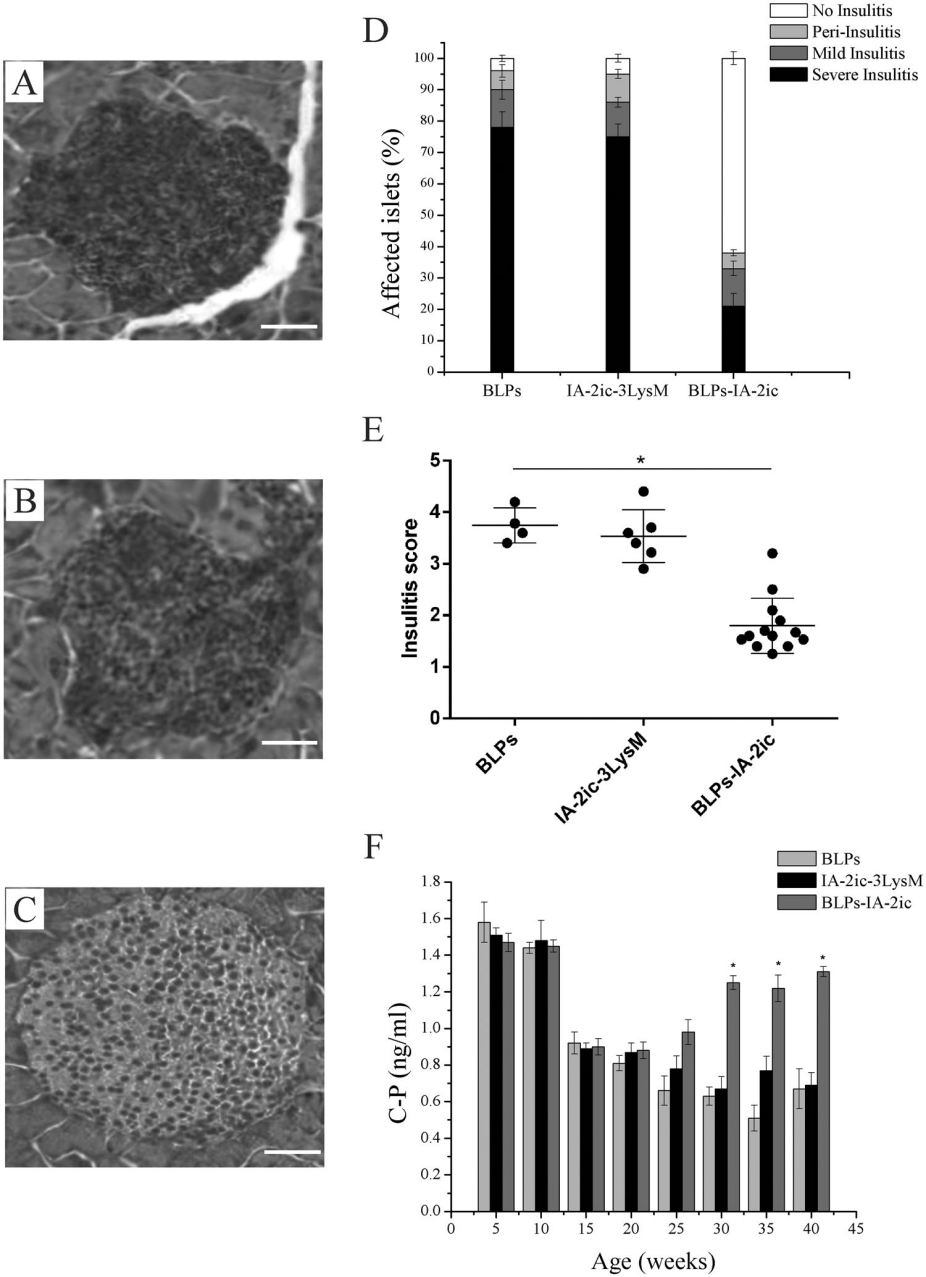
Reduction of insulitis and preservation of C-peptide secretion by oral vaccination with BLPs-IA-2ic. Representative HE staining images of mouse pancreas from BLPs group (A), IA-2ic-3LysM group (B) and BLPs-IA-2ic group (C) at the end of the observation period (40-week-old). Scale bar: 100 μm. (D, E) Comparative analysis of insulitis among groups. All mice alive at the end of the observation period (40-week-old) were tested (*n* = 4 in BLPs group, *n* = 6 in IA-2ic-3LysM group, *n* = 13 in BLPs-IA-2ic group). At least 20 islets per mouse were analyzed. (F) Monitoring of serum C-peptide levels among all groups. For dot plots, each dot represents one mouse. Data are shown as means ± SD. *p < .05.

To clarify the potential mechanism responsible for preventing T1DM, the effect of oral vaccination with BLPs-IA-2ic on suppression of insulitis was analyzed and scored by HE staining at the end of the observation period (40-week-old). Compared with the BLPs group (Figure 6(A)) and IA-2ic-3LysM group (Figure 6(B)), there was less islet inflammation in BLPs-IA-2ic group (Figure 6(C)). Approximately, 78% and 75% of pancreatic islets in the BLPs group and IA-2ic-3LysM group exhibited severe insulitis, respectively. In contrast, this corresponding value of BLPs-IA-2ic group was only 21% (Figure 6(D)). Based on the obtained insulitis score (Figure 6(E)), the mice in BLPs-IA-2ic group exhibited a significant reduction in insulitis compared with the mice in IA-2ic-3LysM group (1.78 ± 0.21 vs. 3.59 ± 0.15, *p* < .05). These observations suggest that oral vaccination with BLPs-IA-2ic was useful to maintain islet integrity in NOD mice.

Furthermore, in order to monitor the pancreatic β cell function, the differences in serum C-peptide levels were recorded through the whole observation period and analyzed (Figure 6(F)). During the first half observation period (5–25 weeks), no significant difference in C-peptide levels was detected among groups. However, the mice in BLPs-IA-2ic group performed significantly higher C-peptide levels compared with the mice in BLPs group and IA-2ic-3LysM group during the remaining observation period (30–40 weeks). Therefore, oral administration of BLPs-IA-2ic endowed mice with the ability to maintain C-peptide secretion.

### 3.6. Activation of IA-2-specific Th2-type immune response by oral vaccination with BLPs-IA-2ic

**Figure 7.**
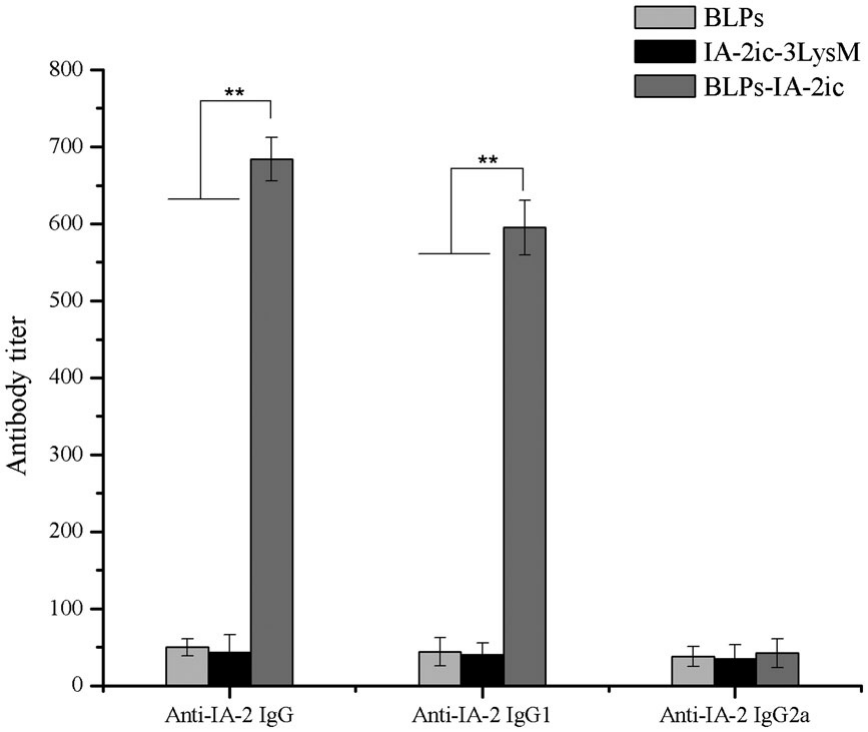
Induction of antigen-specific antibodies by oral vaccination with BLPs-IA-2ic. After the final administration (20-week-old), serum samples collected from all mice were quantified for anti-IA-2 antibodies and antibody subtypes. Data are shown as means ± SD. **p < .01.

After the final administration (20-week-old), serum anti-IA-2 antibody and its subtype levels were measured and analyzed to assess the antigen delivery efficiency of BLPs-IA-2ic as well as to elucidate the mechanism accounting for the suppressive effects on insulitis. As shown in Figure 7, no significant difference was detected in the serum anti-IA-2 IgG levels between the IA-2ic-3LysM group and the negative control group (BLPs group). In contrast, a significant higher anti-IA-2 IgG level was detected in mice in the BLPs-IA-2ic group (*p* < .01). This suggests that BLPs-IA-2ic possessed a high antigen delivery efficiency. The serum isotype levels of T-helper cell type 2 (Th2)-type response-associated IgG1 and Th1-type response-associated IgG2a were measured and used for identifying the type of immune response induced by oral administration of BLPs-IA-2ic. Anti-IA-2 antibodies induced by oral administration of BLPs-IA-2ic were almost exclusively of the IgG1 subclass, as the IgG2a levels among all groups exhibited no significant difference (Figure 7). Therefore, oral administration of BLPs-IA-2ic successfully activated an IA-2-specific Th2-type humoral immune response.

### 3.7. Oral vaccination with BLPs-IA-2ic suppressed splenocyte proliferation in response to IA-2 and induced a Th1 to Th2 cytokine shift

**Figure 8.**
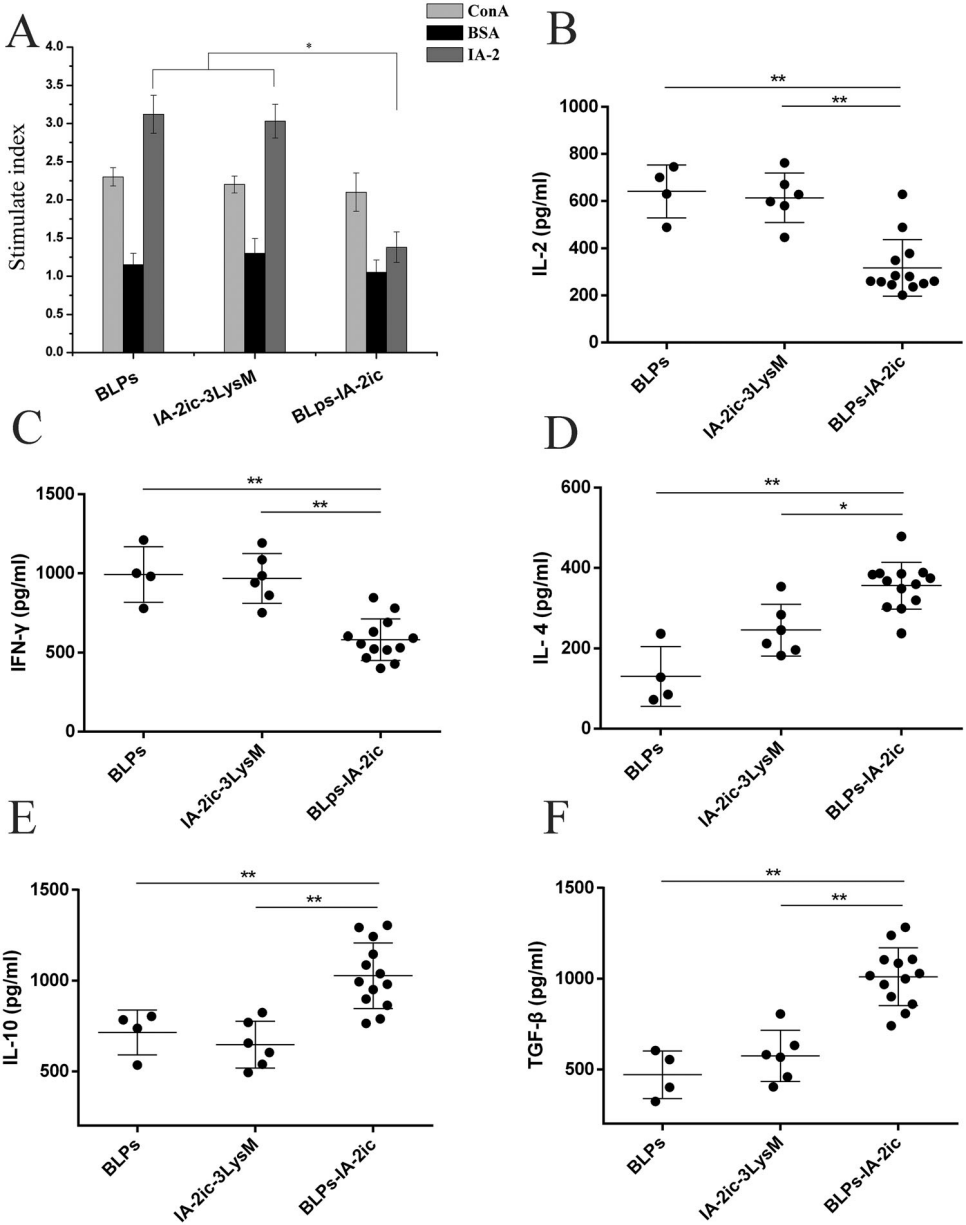
Induction of antigen-specific suppression of splenocyte proliferation and a Th1 to Th2 cytokine shift by oral vaccination with BLPs-IA-2ic. At the end of the observation period (40-week-old), all alive mice in each group (*n* = 4 in BLPs group, *n* = 6 in IA-2ic-3LysM group, *n* = 13 in BLPs-IA-2ic group) were euthanized and subjected to splenocyte proliferation test by stimulating with ConA, BSA, and IA-2 (A) and cytokine analysis in the supernatant of splenic cells after IA-2 stimulation (B–F). For each mouse, at least three independent experiments with three or four biological replicates each were performed. For dot plots, each dot represents one mouse. Data are shown as means ± SD. *p < .05, **p < .01.

Using BSA as a negative control, IA-2 and ConA were used to stimulate splenocytes isolated from NOD mice when the observation period ended (40-week-old), and then the proliferative responses of T cells were presented with SI (Figure 8(A)). Compared with the BLPs group and IA-2ic-3LysM group, there was a significant lower splenocyte proliferation SI in response to IA-2 stimulation in the BLPs-IA-2ic group (*p* < .05). However, all groups exhibited similar reactivations to the positive control ConA and this suggests that T cell reactivity was not suppressed generally by BLPs-IA-2ic vaccination. Therefore, it suggests that the prevention of T1DM in the BLPs-IA-2ic group was associated with the successful downregulation of spontaneous T cell proliferative response to IA-2.

Furthermore, analysis of cytokine secretion by splenocytes stimulated with IA-2 was performed. As shown in Figure 8(B–E), compared to the BLPs group and IA-2ic-3LysM group, splenocytes from the BLPs-IA-2ic group produced significant lower levels of IL-2, IFN-γ (Th1-associated cytokines), and significant higher levels of IL-4, IL-10 (Th2-associated cytokines) (all *p* < .05). TGF-β promotes the differentiation of Tregs and this is a critical step in inducing immunological tolerance to treat autoimmune diseases (Bilate & Lafaille, 2012). A significant higher level of TGF-β was detected in splenocytes from the BLPs-IA-2ic group compared with those from the BLPs group and IA-2ic-3LysM group (Figure 8(F), *p*<.01). Therefore, it indicates that the induced IA-2-specific tolerance by oral administration of BLPs-IA-2ic was associated with downregulation of Th1-associated cytokines and upregulation of Th2-associated cytokines.

### 3.8. Enhancement of Tregs differentiation by oral vaccination with BLPs-IA-2ic

**Figure 9.**
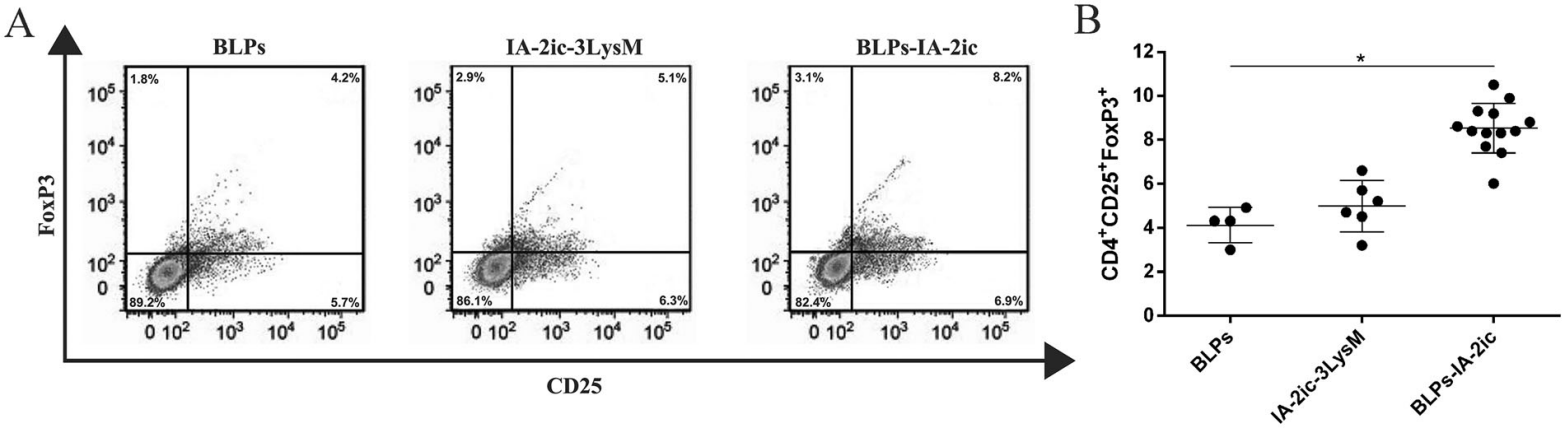
Enhancement of Tregs differentiation by oral vaccination with BLPs-IA-2ic. At the end of the observation period (40-week-old), all alive mice in each group (*n* = 4 in BLPs group, *n* = 6 in IA-2ic-3LysM group, *n* = 13 in BLPs-IA-2ic group) were euthanized and subjected to the analysis of the proportion of CD4^+^CD25^+^FoxP3^+^ Tregs in PLNs by flow cytometry (A), and comparison among groups was analyzed (B). For each mouse, at least three independent experiments with three biological replicates each were performed. For dot plots, each dot represents one mouse. Data are shown as means ± SD. *p < .05.

When the observation period ended (40-week-old), the proportion of CD4^+^CD25^+^FoxP3^+^ Tregs in PLNs was evaluated. A significant higher proportion of CD4^+^CD25^+^FoxP3^+^ Tregs was detected in the BLPs-IA-2ic group compared with those from the BLPs group and IA-2ic-3LysM group (Figure 9, *p*<.05). This indicates that the prevention of T1DM in the BLPs-IA-2ic group was associated with this specifically increased proportions of such Tregs in PLNs.

## 4. Discussion

Current treatment of T1DM mainly focuses on administration of exogenous insulin and improvement of eating habits, in combination with regular physical exercise and sleep. As one of the three broad categories of anticipated emerging and future therapies for T1DM (Powers, 2021), protection of endogenous pancreatic β cells by immunomodulation may be achieved by the T1DM vaccine, which may suppress the onset of autoimmune responses against T1DM-associated autoantigens and/or interrupt these autoimmune responses through the establishment of immunological tolerance (Desai et al., 2021). Induction of antigen-specific immune tolerance by oral vaccination with antigen-based vaccines has been widely tried to delay or prevent the onset of T1DM (Mao et al., 2020a, 2020b). Some studies indicated that a large dose of antigen was needed for the potential therapeutic effect (Bonifacio et al., 2015; Pham et al., 2016). This low efficiency of oral autoantigen therapy may be resulted from the low delivery efficiency of the orally administered autoantigen, which mainly results from the digestion of antigen in the GIT. It has been indicated that LAB BLPs perform a positive role in efficient delivery of an insulin analog to induce oral tolerance to suppress autoimmune diabetes (Mao et al., 2019). In addition, in most cases, the development of autoantibodies to IA-2 is later than that of insulin or GAD65 in the disease progression (Insel et al., 2015). Once the antibody against IA-2 appears as a second or third autoantibody, the risk of the individual reaching the symptomatic T1DM stage (stage 3) increases dramatically (Savola et al., 1998; Katsarou et al., 2017). As a parallel study, the ability of IA-2 to induce oral tolerance and prevent T1DM should be evaluated. Therefore, in the current study, BLPs-IA-2ic vaccine in which the intracellular domain of IA-2 bound to the surface of MG1363 BLPs was constructed and subjected to animal experiments.

As one of the most widely studied and applied heterologous protein expression systems, *E. coli* expression system was used to produce the IA-2ic-3LysM fusion protein. The recombinant proteins are usually expressed in two forms within the *E. coli* host, including the soluble and insoluble forms (Terpe, 2006). Compared with the soluble recombinant proteins existing in the cytoplasm or periplasm, purification of recombinant proteins from the insoluble forms which mainly exist in inclusion bodies is much more complicated, time-consuming, and costly. Therefore, the pET20b(+) expression plasmid containing an N-terminal pelB secretion signal sequence, which can target the recombinant proteins to the periplasm in a soluble form, was used in this study. After purification of His-tagged recombinant proteins with Ni-NTA column, the yield of the soluble IA-2ic-3LysM fusion protein was 46 mg/L fermentation media under the non-optimized induction condition used in this study, which was similar to the yield of recombinant chicken IL-7 (50 mg/L fermentation media) (Cui et al., 2018). In order to reduce the production cost, optimized expression condition and purification approach should be further discovered to increase the IA-2ic-3LysM fusion protein production.

Compared with the living LAB cells, non-living LAB BLPs perform a higher binding capacity for the heterologous protein containing the anchoring domain (Mao et al., 2016). 2.5 × 10^9^ lactococcal BLPs can bear at most 150 μg of the same protein anchor domain containing three LysM repeats used in this study (PA3, 28 kDa) when there was only PA3 added. There were about 10^6^ PA3 molecules per particle under the saturated binding state (Bosma et al., 2006). When the protein anchor fused to the IA-2ic (IA-2ic-3LysM, 66 kDa) in this study, the corresponding binding values were 100 μg and 3.6 × 10^5^ IA-2ic-3LysM molecules. The increased molecular weight as well as protein volume may account for this reduced binding capacity. Based on the stability analysis of the fusion protein in the simulated gastric juice, BLPs-IA-2ic performed a better stability compared with the free fusion protein, and this indicates that the BLPs may provide protection for IA-2ic passing through the GIT, and this was further verified by the animal experiments performed in this study. Unlike the free IA-2ic-3LysM fusion protein, no significant degradation of IA-2ic contained in BLPs-IA-2ic was detected in 60 days, especially at the room temperature. Therefore, these results indicate that a cold-chain route is not essential for delivery of BLPs-IA-2ic vaccine, and this is more important in third-world countries.

Compared to the negative control (BLPs group), the ability of preventing T1DM by oral vaccination with the free IA-2ic-3LysM fusion protein or BLPs-IA-2ic in NOD mice was evaluated. A same T1DM incidence (87%) was detected in the BLPs group and IA-2ic-3LysM group, and however, there was a significant decrease in that of the BLPs-IA-2ic group (33%). The failure to prevent T1DM by oral administration of free IA-2ic-3LysM may be resulted from the rapid degradation of the administered antigens in the GIT, and therefore, there was insufficient antigens in the gut mucosa to stimulate immune tolerance (Pham et al., 2016; Mowat, 2018). The successful prevention of autoimmune diabetes by BLPs-IA-2ic vaccination confirms the protective effect for the delivered proteins provided by BLPs. Based on whether the bound protein releases from the BLPs or not, there may be two forms of the fusion proteins for passing through the intestinal epithelium. One is the free soluble IA-2ic-3LysM, which is released from the BLPs. The other one is that the BLPs-IA-2ic serves as an intact micro-particle. Of course, both forms may exist at the same time. The different forms may rely on different transported routes to access to the immune system and affecting the following immune reactions (Mowat, 2018; Tordesillas & Berin, 2018; Mao et al., 2020a, 2020b). Therefore, how the antigens delivered by BLPs are absorbed and transported through intestinal epithelial cells remains to be analyzed in future studies.

During the progression of T1DM, pancreatic β cells loss and dysfunction resulting from autoimmune destruction presents as an intermittently relapsing progress, leading to insulin deficiency and hyperglycemia (Katsarou et al., 2017; Mao et al., 2020a, 2020b). Based on the limited evidence from human pathologic specimens, insulitis occurs closer to the onset of clinical signs (Wiberg et al., 2015; Campbell-Thompson et al., 2016). Compared to the BLPs group and IA-2ic-3LysM group, the BLPs-IA-2ic group exhibited significantly reduced insulitis. This might indicate that oral administration of BLPs-IA-2ic possesses an effect on the inhibition of islet autoimmune reactions, and therefore preserving islet integrity. This protective effect could be supported by the analysis of pancreatic β-cell function based on the C-peptide levels (Jones & Hattersley, 2013; DiMeglio et al., 2018). Compared to the BLPs group and IA-2ic-3LysM group, the BLPs-IA-2ic group performed significantly higher C-peptide levels during the late observation period (30–40 weeks). The traditional opinion that pancreatic β cells disappear completely and no endogenous insulin production in longstanding diabetes has been drastically changed based on the detection of persistent C-peptide secretion in individuals with long-term T1DM (Flatt et al., 2021; Perkins et al., 2021). Therefore, the protective effect toward islet cells provided by oral administration of BLPs-IA-2ic might be useful for preserving or restoring pancreatic islet reserve before or at disease onset. Additionally, the ability to maintain C-peptide secretion by oral administration of BLPs-IA-2ic might ameliorate various complications as a result of the potential therapeutic provided by C-peptide (Washburn et al., 2021).

It has been suggested that deletion and/or anergy of effector T-cell subsets and induction of regulation through either immune deviation (in favor of Th2 responses) or Tregs account for the mechanisms of autoantigen-induced oral tolerance (Hirsch & Ponda, 2015; Sricharunrat et al., 2018). In this study, an IA-2-specific Th2-type humoral immune response (higher IgG1 levels) was activated by BLPs-IA-2ic vaccination. In addition, based on the analysis of cytokine secretion by splenocytes stimulated with IA-2, oral administration of BLPs-IA-2ic can upregulate the levels of Th2-associated cytokines and downregulate the levels of Th1-associated cytokines. As the Th1/Th2 differentiation is associated with the local cytokine environment (Kidd, 2003), the high levels of Th2-associated cytokines as well as the low levels of Th1-associated cytokines induced by BLPs-IA-2ic may stimulate more naïve T cells to preferentially differentiate into Th2 cells instead of Th1 cells. Therefore, the imbalance of Th1/Th2 which mediates the development of autoimmune disease (Crane & Forrester, 2005) may be repaired by oral vaccination with BLPs-IA-2ic. Conversely, no similar immune response was detected in the BLPs group and IA-2ic-3LysM group, thus accounting for their failure to protect against T1DM. Two different populations of Tregs can be induced by oral vaccination with antigens, including CD4^+^CD25^+^FoxP3^+^ Tregs and Th3 cells, and studies have shown that CD4^+^CD25^+^FoxP3^+^ Tregs are mandatory for oral tolerance (Wawrzyniak et al., 2017). In this study, oral vaccination with BLPs-IA-2ic significantly enhanced CD4^+^CD25^+^FoxP3^+^ Tregs frequency in PLNs. In addition, TGF-β, which exhibits a beneficial role in preventing T1DM by inducing FoxP3 expression and differentiation of peripheral Tregs (Bilate & Lafaille, 2012; Lu et al., 2020), was upregulated by oral vaccination with BLPs-IA-2ic. Overall, the mechanisms responsible for the protection against T1DM by oral vaccination with BLPs-IA-2ic may rely on immune deviation to a Th2 phenotype and induction of CD4^+^CD25^+^FoxP3^+^ Tregs.

## 5. Conclusions

In this study, BLPs-IA-2ic vaccine was successfully constructed based on the heterologous expression and purification of IA-2ic-3LysM fusion protein. Oral vaccination with BLPs-IA-2ic can induce IA-2-specific immune tolerance and suppress autoimmune diabetes in animals. This beneficial effect relies on the repaired Th1/Th2 imbalance by an enhanced regulatory immune response. Thus, oral vaccination with BLPs-IA-2ic serves as a potential strategy for preventing autoimmune diabetes.
